# Comprehensive Analysis of N6-Methyladenosine Regulators in the Subcluster Classification and Drug Candidates Prediction of Severe Obstructive Sleep Apnea

**DOI:** 10.3389/fgene.2022.862972

**Published:** 2022-04-26

**Authors:** Niannian Li, Zhenfei Gao, Jinhong Shen, Yuenan Liu, Kejia Wu, Jundong Yang, Shengming Wang, Xiaoman Zhang, Yaxin Zhu, Jingyu Zhu, Jian Guan, Feng Liu, Shankai Yin

**Affiliations:** ^1^ Department of Otolaryngology Head and Neck Surgery & Center of Sleep Medicine, Shanghai Jiao Tong University Affiliated Sixth People’s Hospital, Shanghai, China; ^2^ Shanghai Key Laboratory of Sleep Disordered Breathing, Shanghai, China; ^3^ Otolaryngology Institute of Shanghai Jiao Tong University, Shanghai, China; ^4^ Department of Medicine, Jiangsu University, Zhenjiang, China

**Keywords:** obstructive sleep apnea, RNA methylation, immunity, consensus clustering, pharmacological intervention

## Abstract

**Background:** Obstructive sleep apnea (OSA) is the most common type of sleep apnea that impacts the development or progression of many other disorders. Abnormal expression of N6-methyladenosine (m6A) RNA modification regulators have been found relating to a variety of human diseases. However, it is not yet known if m6A regulators are involved in the occurrence and development of OSA. Herein, we aim to explore the impact of m6A modification in severe OSA.

**Methods:** We detected the differentially expressed m6A regulators in severe OSA microarray dataset GSE135917. The least absolute shrinkage and selection operator (LASSO) and support vector machines (SVM) were used to identify the severe OSA-related m6A regulators. Receiver operating characteristic (ROC) curves were performed to screen and verify the diagnostic markers. Consensus clustering algorithm was used to identify m6A patterns. And then, we explored the character of immune microenvironment, molecular functionals, protein-protein interaction networks and miRNA-TF coregulatory networks for each subcluster. Finally, the Connectivity Map (CMap) tools were used to tailor customized treatment strategies for different severe OSA subclusters. An independent dataset GSE38792 was used for validation.

**Results:** We found that HNRNPA2B1, KIAA1429, ALKBH5, YTHDF2, FMR1, IGF2BP1 and IGF2BP3 were dysregulated in severe OSA patients. Among them, IGF2BP3 has a high diagnostic value in both independent datasets. Furthermore, severe OSA patients can be accurately classified into three m6A patterns (subcluster1, subcluster2, subcluster3). The immune response in subcluster3 was more active because it has high M0 Macrophages and M2 Macrophages infiltration and up-regulated human leukocyte antigens (HLAs) expression. Functional analysis showed that representative genes for each subcluster in severe OSA were assigned to histone methyltransferase, ATP synthesis coupled electron transport, virus replication, RNA catabolic, multiple neurodegeneration diseases pathway, et al. Moreover, our finding demonstrated cyclooxygenase inhibitors, several of adrenergic receptor antagonists and histamine receptor antagonists might have a therapeutic effect on severe OSA.

**Conclusion:** Our study presents an overview of the expression pattern and crucial role of m6A regulators in severe OSA, which may provide critical insights for future research and help guide appropriate prevention and treatment options.

## Introduction

Obstructive sleep apnea (OSA), a common chronic and complicated disorder, is characterized by repeated complete (apnea) or partial collapse of the upper airway (hypopnea) during sleep, resulting in desaturation and recurring arousal (Peppard et al., 2013). There are over 10% of the population in the world suffering from OSA and will become more prevalent as the population ages. In commonly, an apnea-hypopnea index (AHI) of 30 h a day during sleep or greater is defined as severe OSA. In addition to the obvious clinical symptoms, severe OSA will also increase the risk of adverse medical consequences including cardiovascular disease, diabetes mellitus, dementia, depression, and even cancer (Cortese et al., 2015; Javaheri et al., 2017; Whelton et al., 2018), which impose a huge medical and socioeconomic burden. An increasing number of studies have begun to reveal the potential pathogenesis of OSA, such as upper airway collapsibility (Gleadhill et al., 1991), low arousal threshold (Younes, 2008), inflammation (McNicholas, 2009), immune (Hamada et al., 2017), oxidative stress (Zhou et al., 2016), and so on. However, the exact underlying mechanism of OSA remains to be fully clarified. So far, nocturnal pressure support such as continuous positive airway pressure (CPAP) has been the gold standard for OSA treatment (Sullivan et al., 1981). Although OSA and its adverse effects are effectively alleviated with CPAP, many patients find it intolerable (Mehrtash et al., 2019; Perger and Taranto-Montemurro, 2021), which made pharmacotherapy for OSA a high priority. Collectively, there remains a need for a comprehensive understanding of the pathophysiological mechanisms for OSA and uncovering an alternative pharmacological intervention.

Besides DNA methylation and histone modification being widely known epigenetic mechanisms, post-transcriptional RNA modifications, the crucial regulators of gene expression, have attracted even more and more attention (Roundtree et al., 2017). To date, over 150 unique modifications of RNA have been found in all RNA species, including 5-methylcytosine (m5C), N1-methyladenosine (m1A), Alternative Polyadenylation (APA), and N6-methyladenosine (m6A). Among them, m6A is the most prevalent. M6A modification is a methylated modification formed by methyltransferase complex (MTC) methylation of the sixth position N of adenine on mRNA (Deng et al., 2018), as is well known. RNA binding proteins called “readers” are responsible for recognizing the sites of m6A modification, methyltransferases called “writers” involve in the production of m6A modification while methyltransferases called “erasers” can remove m6A modification (Tong et al., 2018; Chen X.-Y. et al., 2019; Zhang et al., 2021), they work together to keep m6A modification in a dynamic balance. Growing evidence suggests that m6A methylation critically regulates mRNA stability, splicing and protein translation, which in turn impacts gene expression and pathological processes of several human disorders, such as myocardial infarction, myocardial hypertrophy, inflammatory diseases and tumors (Mathiyalagan et al., 2019) (Karthiya and Khandelia, 2020). Researchers have explored the epigenetic mechanisms underlying OSA but they mainly focused on histone modification and DNA methylation mechanisms (Marin et al., 2014; Chen YC. et al., 2019). Recently, Yinghui Chao showed that intermittent hypoxia, a characteristic feature of OSA, promotes the expression of ALKBH5 in lung adenocarcinoma (Chao et al., 2020). A large-scale genome-wide association study had found that rs9937053 near FTO is associated with OSA (Strausz et al., 2021). Despite the fact that the above findings suggest a new view on the mechanism of m6A modification in OSA, the exact role of m6A regulators in OSA remains largely unknown.

In this article, for the first time, we provide a systematic investigation exploring the role of m6A epigenetic regulation in severe OSA. We found that IGF2BP3 can be used to well distinguish severe OSA patients from controls in both the training set and validation set. Furthermore, we revealed three distinct severe OSA subclusters with different immune infiltration characteristics. In addition, functional analysis, protein-protein interaction (PPI) networks and miRNA-TF coregulatory networks were performed to uncover the biological functions for each subcluster. Finally, Connectivity Map (CMap) tools were used to search for small molecular compounds that could potentially reverse the altered expression of differentially expressed genes (DEGs) in different clusters, which could provide novel insights for future clinical therapies or adjuvant treatment of OSA.

## Materials and Methods

### Data Collection

**FIGURE 1:**
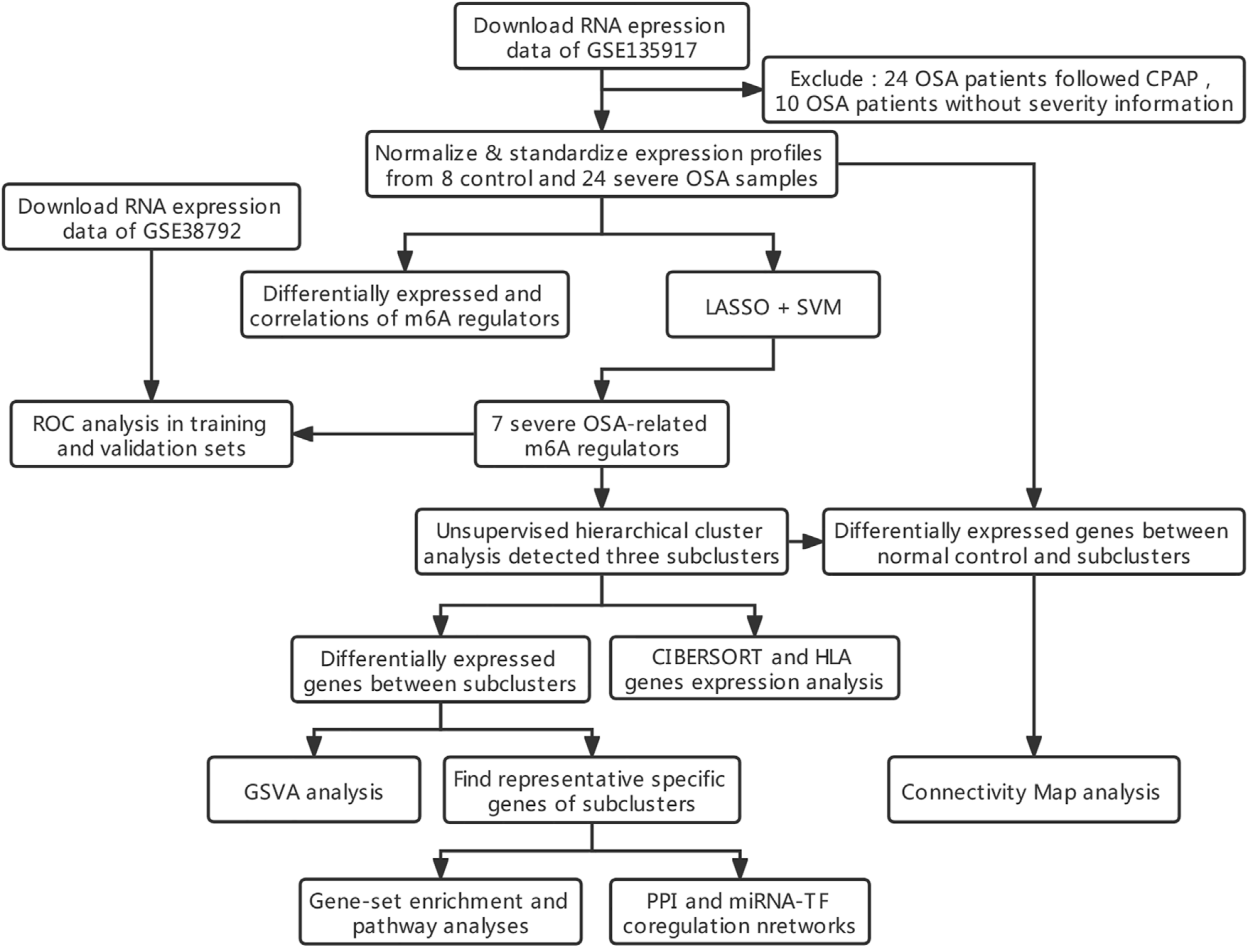
Workflow and analysis strategy used in the current study. Abbreviations: LASSO, least absolute shrinkage and selection operator. SVM, support vector machine. ROC, receiver operating characteristic curve. GSVA, gene set variation analysis. GO, Gene Ontology. KEGG, Kyoto encyclopedia of genes and genomes. PPI, protein-protein interaction networks. TF: transcription factor.

The mRNA expression profiling datasets GSE135917 and GSE38792 represent interchange of gene expression in OSA were downloaded from the GEO database (Barrett et al., 2007) by using the R package “GEOquery” (Davis and Meltzer, 2007) (version 2.60.0). GSE135917 (Gharib et al., 2020)was used as a training dataset which contains 66 subcutaneous adipose tissue samples totally. Among them, 24 patients follow CPAP might affect the transcriptome profiles and 10 individuals with unknown severity of OSA were excluded, finally, eight healthy controls and 24 patients with severe OSA were included for analysis. GSE38792 (Gharib et al., 2013) consists of eight normal subjects and 10 OSA visceral fat tissue samples were used for validation. GPL6244 (Affymetrix Human Gene 1.0 ST Array) is used for both datasets. We normalized the expression profiles, and then standardize the data through the “normalizeBetweenArrays” function of the “limma” package (Ritchie et al., 2015) (version 3.48.3), so that the expression value has a similar distribution (Supplementary Figure S1). Gene probes were annotated with official gene symbol, and mean values were taken if multiple gene probes matched to the same gene. The workflow of this study is shown in Figure 1.

### Alternations of m6A Regulators Between Severe Obstructive Sleep Apnea and Healthy

The 23 selected m6A regulators were involved in our study, including 8 writers (CBLL1, METTL14, METTL3, ZC3H13, RBM15B, WTAP, RBM15 and KIAA1429), 13 readers (YTHFDF1, YTHDF2, YTHDF3, YTHDC1, YTHDC2, HNRNPC, HNRNPA2B1, IGF2BP1, IGF2BP3, FMR1, ELAVL1 and LRPPRC) and two erasers (FTO and ALKBH5) (Zhang et al., 2020). We used the Wilcox test to compare the expression differences of 23 m6A regulatory factors between OSA patients and controls. Spearman correlation analysis was used to determine the correlation between m6A regulators with the R package “corrplot” (Wei et al., 2013) (version 0.91). Moreover, two widely available machine learning algorithms, LASSO and SVM, were used to identify the severe OSA-related m6A regulators. LASSO uses regularization to solve the over fitting in the process of curve fitting and improve the accuracy of the model. We use the “glmnet” package (Simon et al., 2011) (version 4.1–2) to build the model with m6A regulators. SVM is a supervised machine learning algorithm based on the structural risk minimization principle from statistical learning theory. In our research, the “e1071” (Meyer et al., 2015) (version 1.7–9) package was used to plot every data point as a dot in n-dimensional spaces (where n is the number of the m6A regulators) and find an optimal hyperplane that differentiates the two classes (non-OSA and severe OSA) very well. The severe OSA-related m6A regulators obtained by the results of two algorithms overlapped. And then, the R package “pROC” (Robin et al., 2011) (version 1.18.0) was used to calculate the area under the curve (AUC) and evaluate the distinguishing performance of m6A regulators.

### Three Distinct M6A Patterns Identified by Severe Obstructive Sleep Apnea-Related m6A Regulators

Consistent clustering is commonly used to detect disease-related molecular subtypes and determine the number of possible cluster members in view of gene expression profiles. Unsupervised consensus clustering was conducted to classify OSA into different groups based on the expression profiles of seven severe OSA-related m6A regulators by using the “ConsensuClusterPlus” (Wilkerson and Hayes, 2010) (version 1.56.0) package. In this process, the parameters were pam algorithm with the spearman distance and sampling was repeated for 1,000 times to make the stratification more stable. The number of clusters was identified through the cumulative distribution function. “Rtsne” (Van and Hinton, 2008) (version 0.15) package was used to display the distribution of different subcluster samples. The m6A regulator expression among the three distinct modification patterns were compared by the Kruskal-Wallis test.

### Estimation of Immune Characteristics of Different m6A Patterns

CIBERSORT is a widely used algorithm that uses support vector regression modeling to deconvolute cell types and has been applied to several diseases (Newman et al., 2019). We used CIBERSORT to profile 22 immune cell types (T cells CD8, T cells CD4 naïve, T cells CD4 memory resting, T cells CD4 memory activated, B cells naïve, B cells memory, NK cells resting, NK cells activated, macrophages M0, macrophages M1, macrophages M2, dendritic cells resting, dendritic cells activated, mast cells resting, mast cells activated, neutrophils and eosinophils) in severe OSA patients based on the expression matrix. The infiltrating immunocyte abundance score and HLA genes expression among three distinct m6A patterns were compared by the Kruskal-Wallis test. *p* < 0.05 was considered statistically significant.

### Differential Expression Analysis of Genes Among Different m6A Patterns

The “limma” package was used to identify the DEGs. Cut-off criteria was obtained between normal group and severe OSA subclusters using adjusted *p*-value<0.05 and absolute value of log2-fold change >1. Additionally, *p* < 0.05 was used as the threshold value for DEGs between different subclusters. The representative genes for each OSA subclusters were determined by a Venn plot method. For example, to determine the representative genes of cluster1, a Venn plot of (cluster1 vs. cluster2) vs. (cluster1 vs. cluster3) was drawn to get the overlap of DEGs.

### Biological Enrichment Analysis Among Different m6A Patterns

GSVA (gene set variation analysis) is an unsupervised enrichment analysis method to evaluate the pathway activity variation. By transforming the genes expression matrix of different subjects into the matrix of pathway activation score, we can evaluate whether different metabolic pathways are enriched in different OSA subclusters by“GSVA” (Hänzelmann et al., 2013) (version 1.40.1) and “limma” packages. The gene set of “c2.all.v7.4.symbols”was downloaded from the MSigDB database for running GSVA analysis and *p* < 0.01 was regarded as the threshold of the differential analysis. We visualized the significantly changed pathways among OSA subclusters with the R package “pathview” (Luo and Brouwer, 2013) (version 1.32.0), which can visualize metabolic pathways using predefined layout maps for a subset of reactions. To provide a deeper understanding of the biological functions of the representative genes for each OSA subcluster, we performed Kyoto Encyclopedia of Genes and Genomes (KEGG) pathway enrichment and gene ontology (GO) analysis with R package “clusterProfiler” (Yu et al., 2012) (version 4.0.5). The parameter “ont” was set to “all,” which included biological process (BP), molecular function (MF) and cellular component (CC) categories. The functional analysis was set to adjust the *p*-value <0.05 as the cut-off criterion.

### Construction of Protein-Protein Interaction Network and MiRNA-TF Coregulatory Network

Search Tool for the Retrieval of Interacting Genes (STRING)(Szklarczyk et al., 2019) is a database that searches for the interaction between known proteins and predicted proteins. It delivers the results obtained from experimental data, text mining from PubMed summary and predicted by bioinformatics methods. Representative genes for each subcluster were inserted in the STRING for generating the PPI network and the confidence score was set at 0.8. PPIs were exported from the STRING database and then entered into Cytoscape for a better visual representation and analysis of hub genes. Molecular complex detection (MCODE), a plugin of Cytoscape, is always used to identify highly interconnected parts and help researchers distinguish profound genes. We performed cluster analysis of the PPI network by using MCODE to detect key modules, and genes in the essential module with the highest MCODE score were regarded as hub genes. NetworkAnalyst (Xia et al., 2014; Xia et al., 2015) is a comprehensive website that allows users to undertake gene expression analysis for a variety of species as well as meta-analysis. We used this tool to collect data from the RegNetwork repository (Liu et al., 2015) to build the TF-miRNA coregulatory networks, which help us understand the regulatory mechanism of hub genes at the transcriptional and post-transcriptional level. Moreover, Cytoscape was used to visualize the interaction network among miRNA, TFs and hub genes.

### Compounds Targeting With Different Obstructive Sleep Apnea Subclusters

To determine which molecular compounds might be useful against different OSA subclusters, we used the CMap tools (Lamb et al., 2006). The CMap collected 164 small molecules compounds and four cell lines (PC3, HL60, MCF and SKMEL5) expression profiles, and discussed the interaction network among compounds, genes and disease status. We predict compounds that can activate or inhibit based on the differentially expressed genes between normal group and severe OSA subclusters. Due to the limitation Of the Connectivity Map tool that matches gene symbol and HG-U133A (Malta et al., 2018), we transformed the gene symbol of DEGs into GPL96 platform ID. Moreover, we performed particular analysis to learn more about the mechanism of action (MoA) of the compounds (Subramanian et al., 2017).

### Statistical Analysis

All statistical analyses were performed using R version 4.0.2. The independent-sample *t*-test and Mann–Whitney *U*-test were applied for the comparison between groups as appropriate. Different m6A modification subclusters were compared using the Kruskal-Wallis test. All analyses were based on two-tailed tests, *p* < 0.05 was considered to be statistical significance.

## Results

### The Landscape of m6A Regulators and Its Dysregulated Expression in Severe Obstructive Sleep Apnea

**FIGURE 2:**
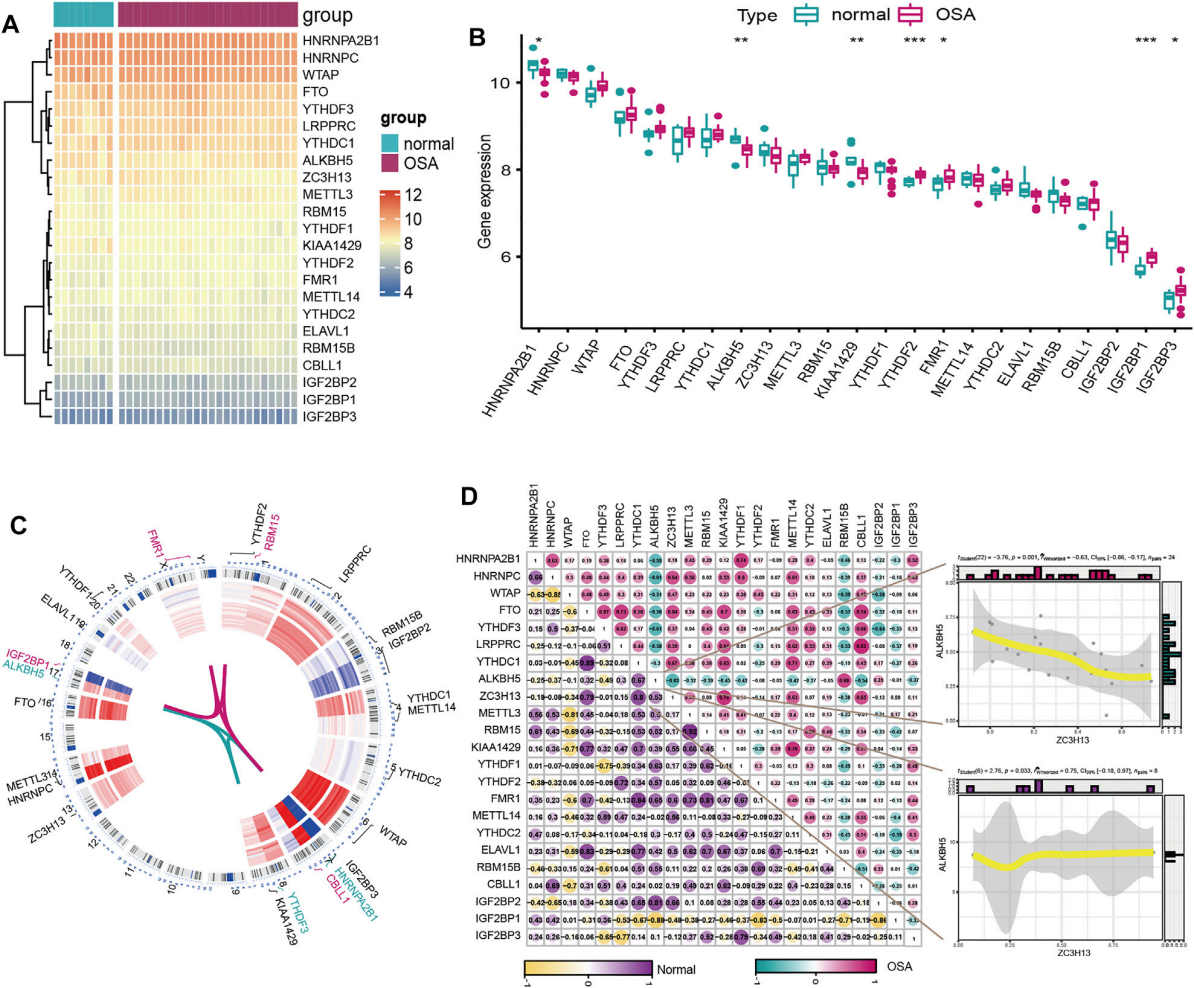
The expression pattern of m6A regulators in severe OSA and control individuals based on the GSE135917 dataset. **(A)** Heatmap and **(B)** boxplot of 23 m6A genes expression between normal and severe OSA subjects. **(C)** Chromosomal positions and expression of the 23 m6A regulators. **(D)** Correlations of 23 m6A regulators in normal and OSA samples. The two scatter plots demonstrated the changes of correlations of ZC3H13 and ALKBH5 between severe OSA and normal subjects.

We explored the expression differences of the selected 23 m6A regulators between healthy and severe OSA patients, and the results revealed that seven m6A regulators were significantly dysregulated. Specifically, the expression of HNRNPA2B1, KIAA1429 and ALKBH5 were lower in severe OSA patients than in normal controls, while YTHDF2, FMR1, IGF2BP1 and IGF2BP3 were upregulated (Figures 2A,B). And we also visualized the chromosomal position of the 23 m6A regulators (Figure 2C). To explore whether m6A regulators functioned crucially in the development of the severe OSA, we evaluated correlations between m6A regulators based on transcriptome profiles in severe OSA and controls separately. Interestingly, the correlation of m6A regulators between two groups has a significant change (Figure 2D). RBM15 and METTL3 are the most correlated m6A regulators in normal group (with correlation R values of 0.92) but the correlation is weak in severe OSA patients (with correlation R values of 0.14). In addition, ZC3H13 and ALKBH5 were positively correlated in controls (with correlation R values of 0.53) but negatively correlated in severe OSA (with correlation R values of −0.63) (Figure 2D), implying that m6A regulators played a major role in the development of severe OSA.

### Identification of the Severe Obstructive Sleep Apnea-Related m6A Regulators

**FIGURE 3:**
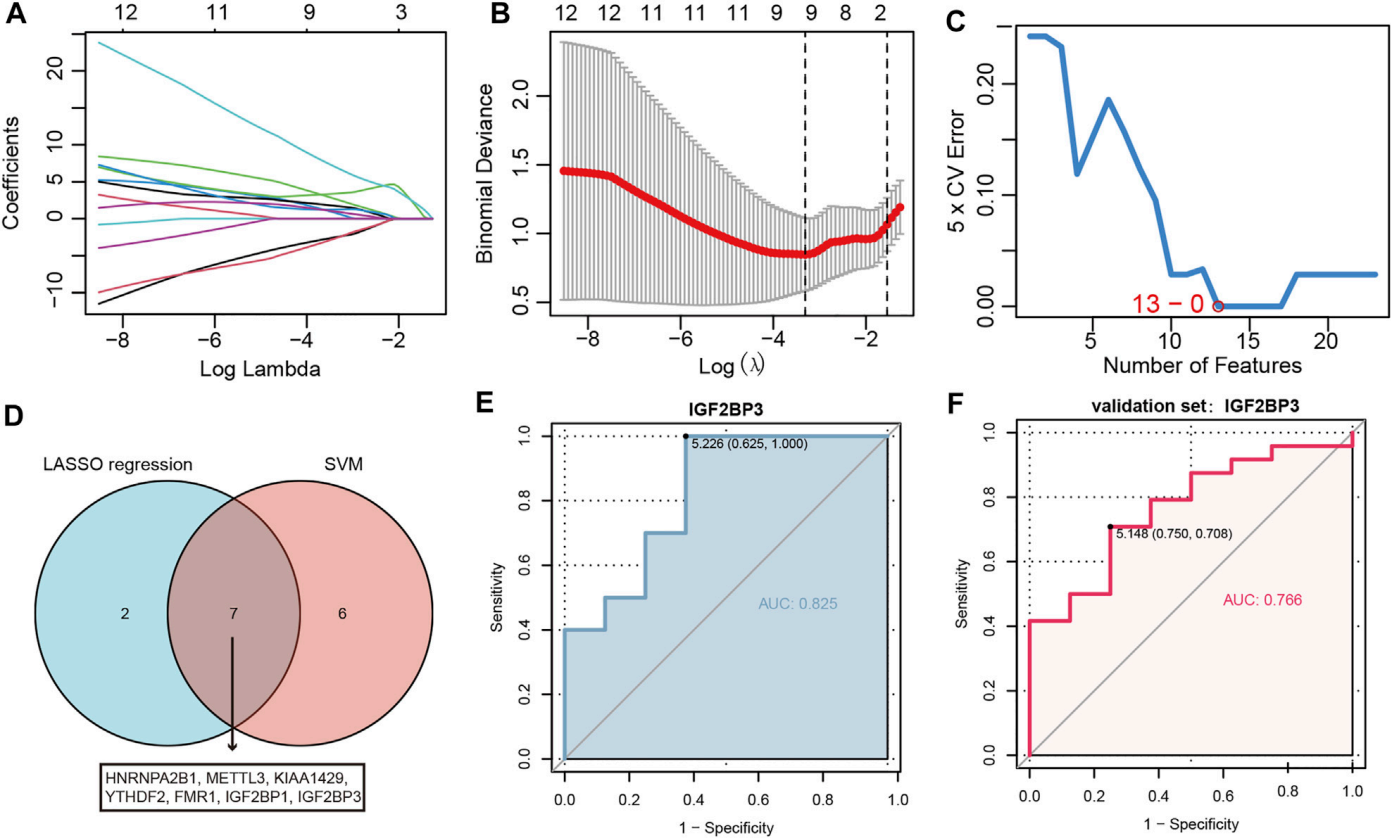
IGF2BP3 can well distinguish severe OSA from normal individuals. **(A)** LASSO coefficient profiles of m6A regulators. **(B)** 10-fold cross-validation for tuning parameter selection in the LASSO regression. **(C)** The point highlighted indicates the lowest error rate, and the corresponding m6A regulators at this point are the best signature selected by SVM. **(D)** Venn diagram demonstrating seven OSA-related genes shared by the LASSO and SVM algorithms. **(E,F)** The discrimination ability of IGF2BP3 was evaluated by ROC curve and AUC value based on training set GSE135917 **(E)** and validation set GSE38792 **(F)**. AUC: area under the curve.

A series of machine learning methods were used to screen severe OSA-related m6A regulators. We performed LASSO regression on the 23 m6A regulators for feature selection and dimension reduction (Figures 3A,B), 9 m6A regulators were found to be important for OSA. At the same time, SVM was used to find the best variables by deleting SVM-generated Eigenvectors, and 13 m6A regulators were identified (Figure 3C). The severe OSA-related m6A regulators were obtained by the results of two algorithms overlapped (Figure 3D), and finally seven m6A regulators remained, including HNRNPA2B1, METTL3, KIAA1429, YTHDF2, FMR1, IGF2BP1 and IGF2BP3. To further test the diagnostic efficacy of these m6A regulators, we performed ROC analysis and validated it with the GSE38792 dataset. The results illustrated IGF2BP3 can be used to well distinguish severe OSA patients from normal healthy (AUC = 0.825) (Figure 3E) and also reached a higher level in the validation set (AUC = 0.766) (Figure 3F), indicating that IGF2BP3 had a high diagnostic value.

### Distinct m6A RNA Methylation Modification Patterns Identified by the Severe Obstructive Sleep Apnea-Related m6A Regulators

**FIGURE 4:**
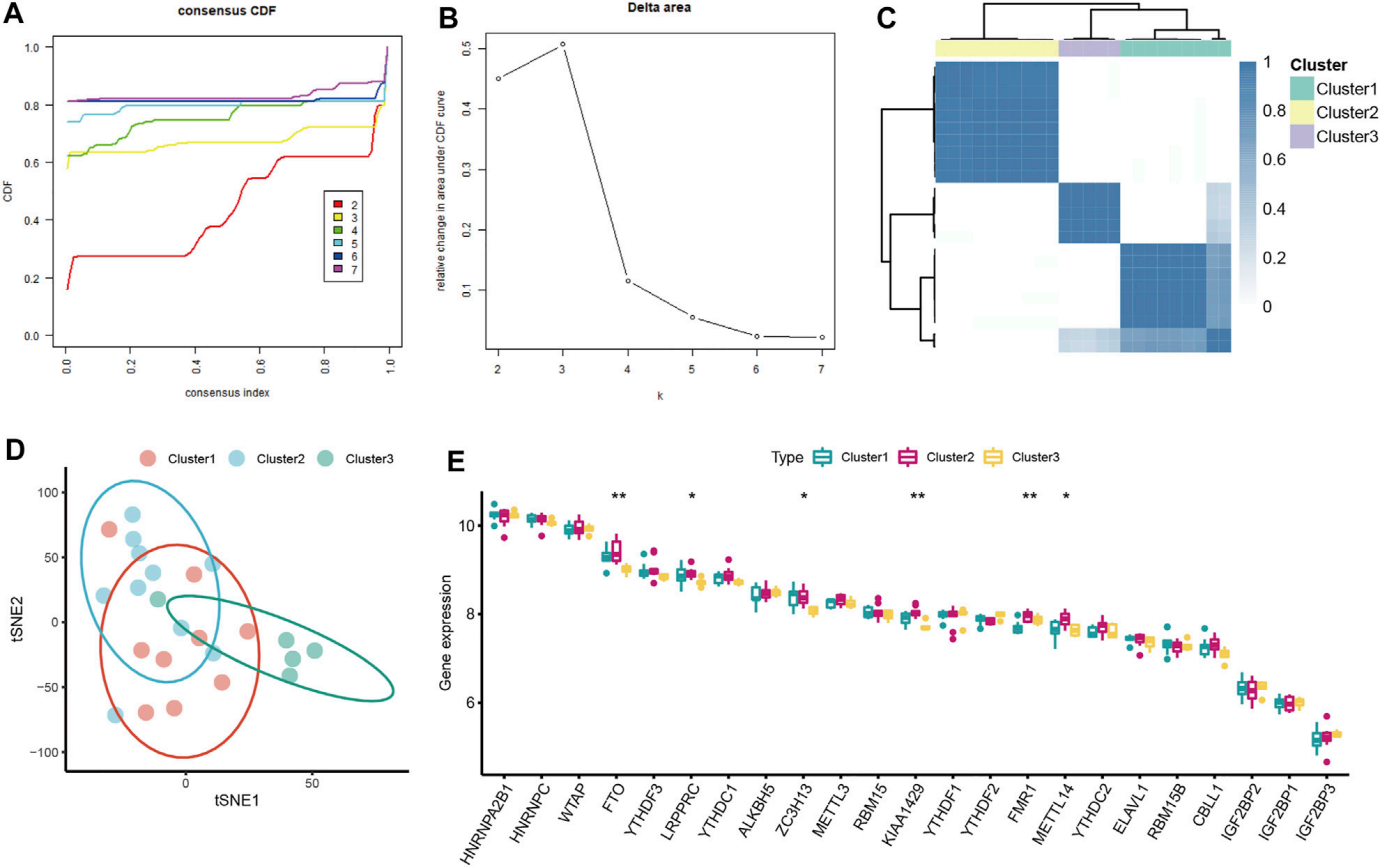
Consensus clustering analysis of severe OSA subjects based on mRNA levels of OSA-related m6A regulators. **(A)** Consensus clustering cumulative distribution function (CDF) for k = 2–7. **(B)** Relative change in area under CDF curve fork = 2–7. **(C)** OSA subjects were divided into three clusters when k = 3. **(D)** The tSNE plot of the transcriptome profiles of 3 m6A subtypes. **(E)** The different expression status of 23 m6A regulators among three m6A subtypes.

Unsupervised consensus clustering analysis was conducted to identify distinct m6A patterns based on the expression of seven severe OSA-related m6A regulators. Three distinct subclusters of severe OSA were identified (Figures 4A–C), including 9 cases in cluster1, 10 cases in cluster2, and 5 cases in cluster3 (Figure 4D). We explored the general expression pattern of m6A regulators to uncover the differences among the three subclusters. The result showed that FTO, LRPPRC, ZC3H13, KIAA1429, METTL14 were significantly downregulated in cluster3 (Figure 4E), FMR1 expression level was lowest in cluster1, validating the existence of diversity m6A modification patterns in severe OSA.

### Infiltration Characteristics of Immune Microenvironment Among Distinct m6A Modification Patterns

**FIGURE 5:**
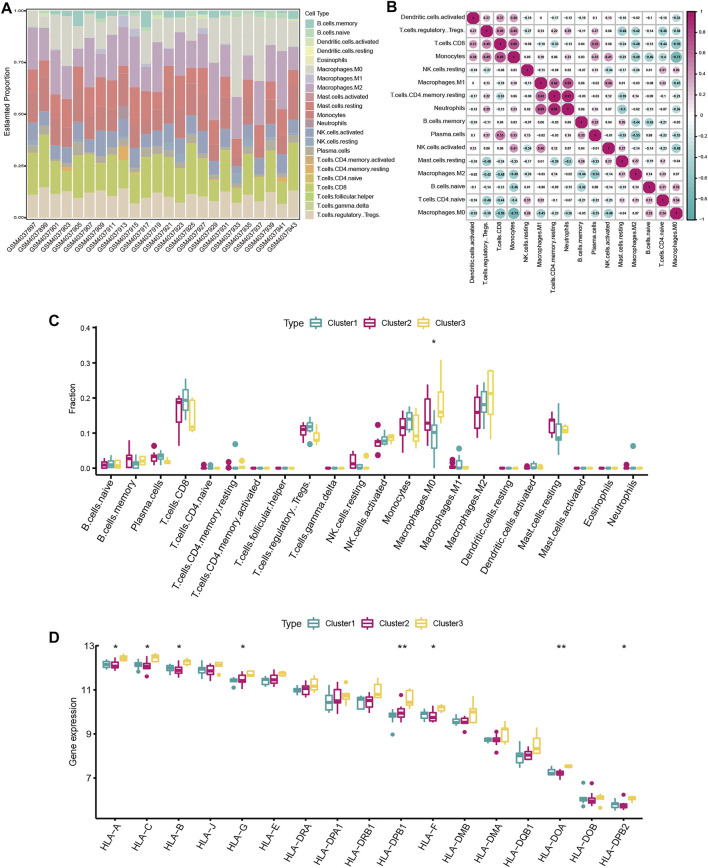
Immune microenvironment characteristics among distinct severe OSA subtypes. **(A)** Relative proportions of immune cell infiltration in severe OSA individuals. **(B)** Correlation matrix of the 22 immune cell proportions. **(C)** Differences in immune cell infiltration abundances among three m6A modifications. **(D)** The expression differences of each HLA gene in three m6A modification patterns.

In order to explore the differences in immune microenvironment characteristics, the CIBERSORT algorithm was used to evaluate infiltrating immunocytes among different subclusters. The relative proportion of immune cell subtypes of different individuals was displayed in the stacked barplot (Figure 5A) and boxplot (Figure 5B). The infiltration level of M0 Macrophages had great variation among three subclusters (Figure 5C). M0 Macrophages are enriched most in cluster3, while least in cluster2. Although not statistically significant, we observed a higher infiltrated level of M2 Macrophages but a lower infiltrated level of T cells in cluster3, suggesting that patients in cluster3 have a potential regulatory role in macrophage polarization. As for HLA genes expression, HLA-A, HLA-B, HLA-C, HLA-G, HLA-DPB1, HLA-F, HLA-DOA, HLA-DPB2 expression level was remarkably highest in cluster3 (Figure 5D), suggesting patients in cluster3 might mediate an active immune response. Cluster1 and cluster2 have comparable expression levels of HLA genes. The results showed that there is obvious heterogeneity in severe OSA patients, and m6A regulators had a crucial role in shaping different immune microenvironments in severe OSA.

### Functional Enrichment Analysis of Three m6A Modification Patterns

**FIGURE 6:**
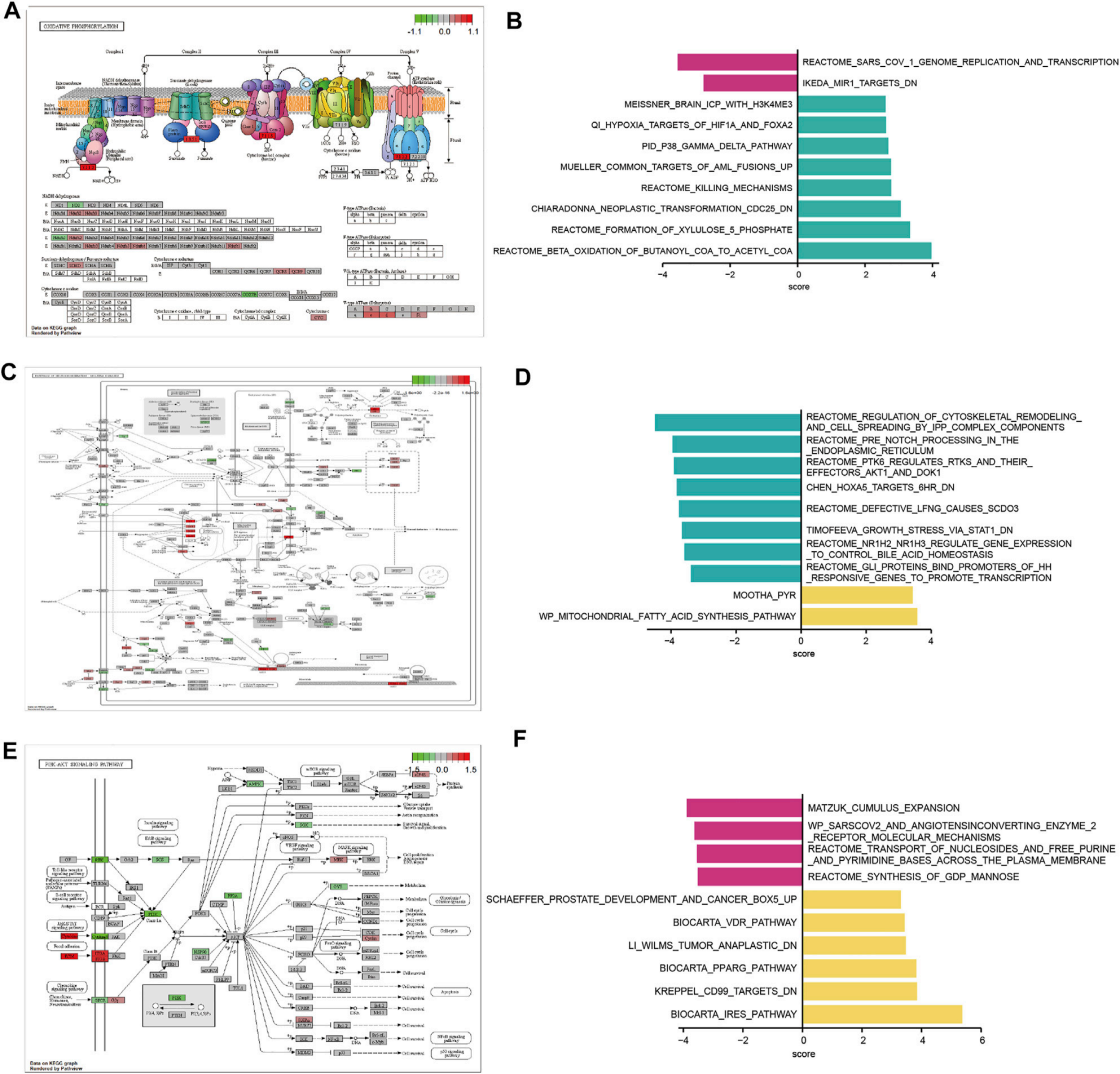
Three m6A modification patterns differ in their underlying biological function characteristics. **(A,C,E)** Integrated KEGG pathway analysis and visualization of both gene expression and metabolomics data. Gene expression levels are indicated as significantly higher (red), unchanged (gray), or lower (green). The differences of hsa00190 Oxidative phosphorylation pathway between m6A modification pattern 1 and pattern 2 **(A)**. The differences of hsa05022: Pathways of neurodegeneration - multiple diseases between m6A modification pattern 1 and pattern 3 **(C)**. The differences of hsa04151: PI3K-Akt signaling pathway between m6A modification pattern 2 and pattern 3 **(E)**. **(B,D,F)** Gene set variation analysis (GSVA) for significantly enriched pathways between subcluster 1 and subcluster 2 **(B)**, subcluster 1 and subcluster 3 **(D)**, subcluster 2 and subcluster 3 **(F)**.

**FIGURE 7:**
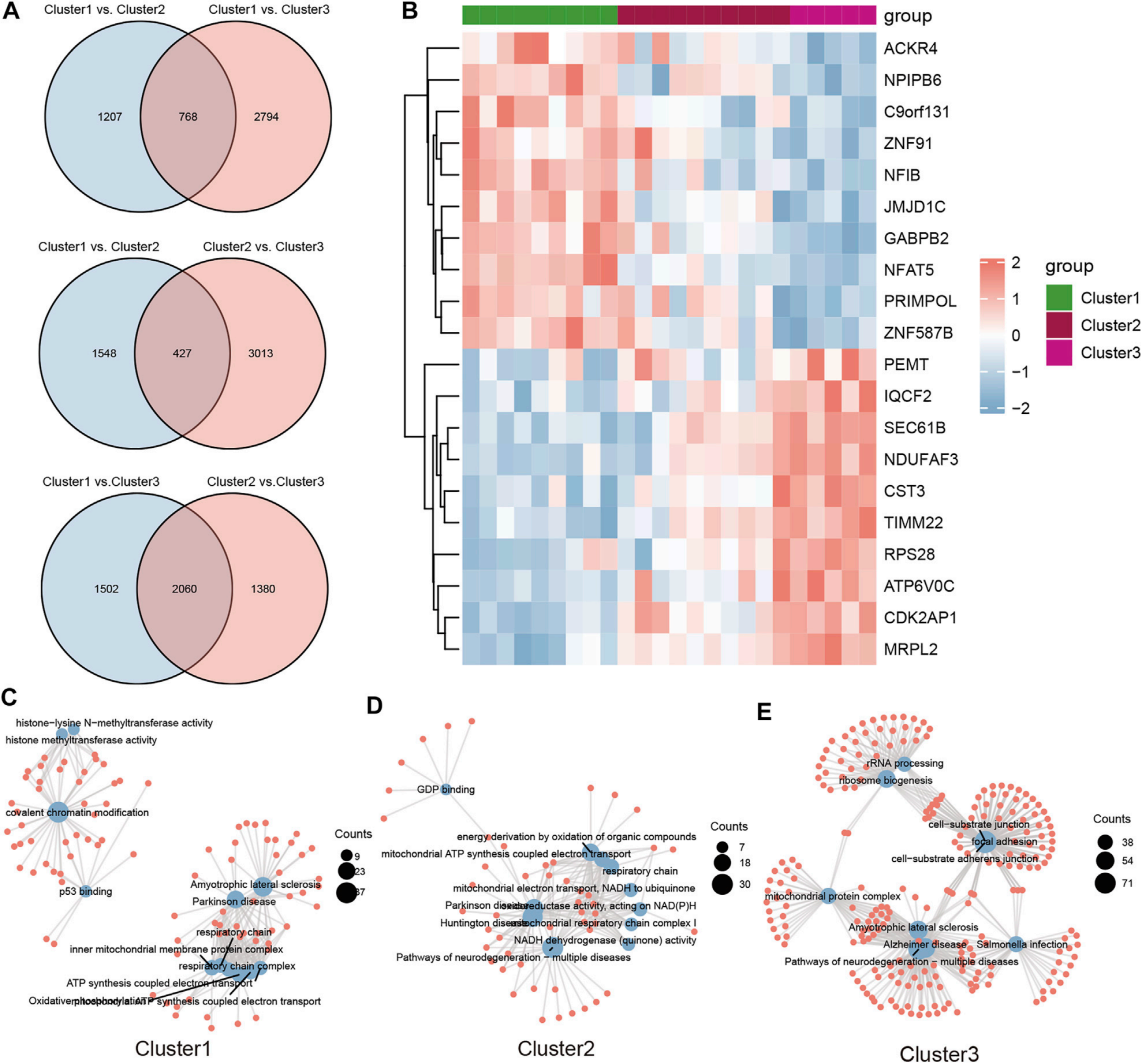
The representative genes and functional analysis for each severe OSA subtype. **(A)** The differential genes were calculated for each of the two subclusters and intersected using the Venn plot. Cluster1, cluster2 and cluster3 consisted of 768, 427 and 2060 representative genes, respectively. **(B)** Heatmap of top 10 represented genes in all three subtypes. **(C–E)** GO and KEGG analysis of representative genes for each subcluster.

To investigate the biological functions in the three m6A modification patterns, we applied KEGG pathway analysis and GSVA enrichment analysis to reveal significant differential gene sets between severe OSA subtypes. KEGG views of the most enriched pathways were generated, including Oxidative phosphorylation (Figure 6A), Pathways of neurodegeneration—multiple diseases (Figure 6C) and PI3K-Akt signaling pathway (Figure 6E) for each pairwise comparison, respectively. Compared with cluster2, “BETA OXIDATION OF BUTANOYL COA TO ACETYL COA” and “FORMATION OF XYLULOSE 5 PHOSPHATE” pathways were significantly active in cluster1 (Figure 5B). “REGULATION OF CYTOSKELETAL REMODELING AND CELL SPREADING BY IPP COMPLEX COMPONENTS” was inhibited in cluster1 when compared with cluster3 (Figure 6D). “PPARG PATHWAY” was enriched in cluster3 and “SYNTHESIS OF GDP MANNOSE” pathway was inhibited (Figure 6F). To further understand the molecular mechanisms in each OSA pattern, we used the Venn plot to identified the subclusters’ representative genes (Figure 7A). Cluster1, 2, 3 contained 768, 427 and 2060 representative differentially expressed genes, respectively (Figure 7B). GO analysis showed that representative genes from cluster1 mainly related to histone methyltransferase and ATP synthesis coupled electron transport (Figure 7C), and cluster2 involved in mitochondrial energy metabolism (Figure 7D). Cluster3’s representative genes are enriched in RNA catabolic, cell adhesion and mitochondrial protein complex terms (Figure 7E). As we can see, the three m6A modification patterns have both unique and shared biological functions. The common enriched terms of these clusters were mitochondrial ATP synthesis coupled electron transport process and multiple neurodegeneration diseases pathway, suggesting they may result from an early insult that leads to severe OSA.

### Construct Protein-Protein Interaction Network and MiRNA-TF Coregulatory Network for Each m6A Pattern

**FIGURE 8:**
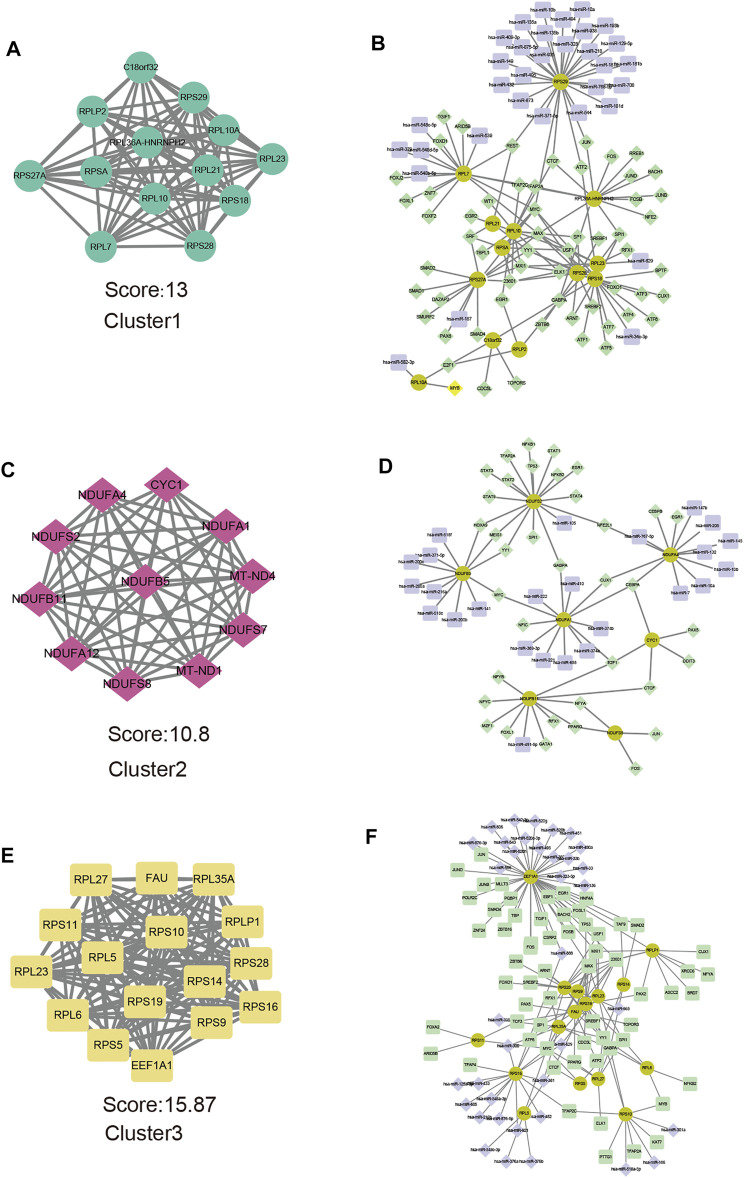
Hub genes analysis and miRNA-TF coregulatory networks of representative genes of each subtype. Modules with the highest MCODE scores from representative genes of cluster1 **(A)**, cluater2 **(C)** and cluster3 **(E)** are illustrated. Networks for hub-genes miRNA-TF interaction with representative genes of cluster1 **(B)**, cluater2 **(D)**, and cluster3 **(F)** are shown. The highlighted yellow color nodes represent the hub genes, purple color nodes represent the miRNA and green nodes represent TF-genes.

Representative genes for each m6A pattern were provided as an input in the STRING and the results were reintroduced into Cytoscape software for visual representation and further module analysis. We identified the key modules and hub genes by using the MCODE plug-in. At the same time, the miRNA-TF coregulatory network was used to reveal gene regulatory networks. In cluster1, there were 263 nodes and 633 edges included in the PPI network (Supplementary Figure S2A), the top 10 genes with the most interactions and the number of protein-protein interaction pairs were shown in the barplot (Supplementary Figure S2B). Hub genes of cluster1 were illustrated in Figure 8A. The miRNA-TF coregulatory network comprised of 105 nodes and 143 edges (Figure 8B), RPS29 enriched of miRNA regulators and was regulated by 24 miRNAs. Transcription factors MYC and MAX hold the key position that connected eight hub genes. In cluster2, there are 139 nodes, 313 edges in the PPI networks (Supplementary Figures S2C, S2D), and 11 hub genes were shown in Figure 8C the miRNA-TF coregulatory network consisted of 68 nodes and 75 edges, among them, NDUFS2 was regulated by 15 transcription factors (Figure 8D). In cluster3, there are 446 proteins and 1,174 protein interaction pairs (Supplementary Figures S2D, S2E), and 16 hub genes were identified (Figure 8E). the miRNA-TF coregulatory network consisted of 115 nodes and 166 edges, where EEF1A1 had the most interactions with these miRNA and transcription regulators (Figure 8F), suggesting it might play an important role in severe OSA cluster3.

### Identifies Potential Compounds Capable of Targeting With Different m6A Patterns

**FIGURE 9:**
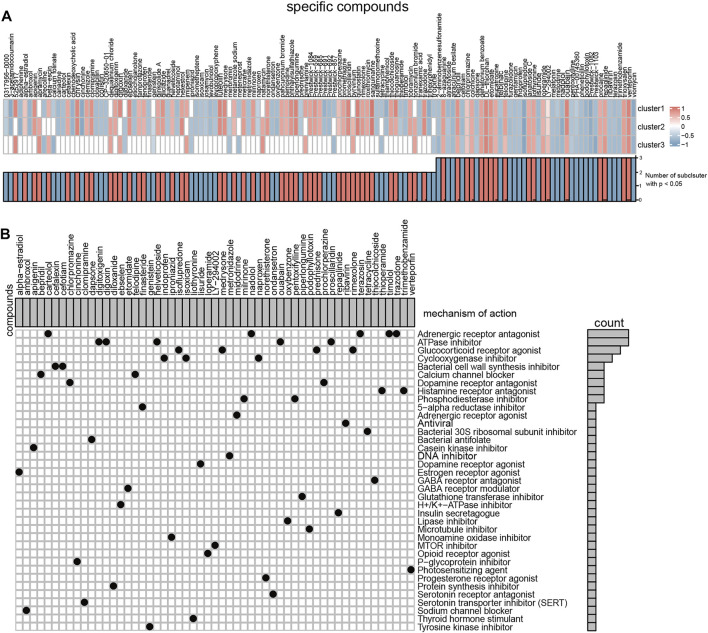
Connectivity Map tools predict the small molecules compounds target different severe OSA subclusters. **(A)** Heatmap showing enrichment score (positive in blue, negative in red) of each compound from the CMap for each OSA subtypes. **(B)** Heatmap showing each compound (perturbagen) from the CMap that shares mechanisms of action (rows) and sorted by descending number of compounds with shared mechanisms of action.

The CMap tools measure the connectivity between disease gene expression signatures and compound-induced gene expression profiles. We detected differentially expressed genes between normal healthy and subclusters (Supplementary Figure S3). And then, CMap was used to find prospective medicines that can effectively reverse the differential gene expression of different severe OSA subclusters. Totally, we found 133 compounds showed a significant impact on the expression profile in at least one severe OSA subtype. 24 compounds were significantly enriched in all three OSA subclusters (Figure 9A), and 43 compounds enriched in two of the subclusters. According to the MoA analysis, the above compounds have 38 mechanisms of action in common (Figure 9B). The results showed that five drugs (carteolol, nadolol, terazosin, timolol and trazodone) shared the MoA of Adrenergic receptor antagonist, another five compounds (digitoxigenin, digoxin, helveticoside, ouabain and proscillaridin) shared the MoA of ATPase inhibitor. We also found indoprofen, isoxicam and naproxen shared the MoA as cyclooxygenase inhibitor, thioperamide, trimethobenzamide share the MoA as histamine receptor antagonists, and bepridil, felodipine shared the MoA as calcium channel blocker. The above ATPase inhibitors and calcium channel blockers can aggravate the expression changes and may worsen the situation in patients with severe OSA, while cyclooxygenase inhibitors and histamine receptor antagonists have a therapeutic effect. Of note, the result also showed that different compounds that act on adrenergic receptors have a different response to severe OSA patients, carteolol, nadolol, terazosin, timolol might be beneficial to severe OSA patients.

## Discussion

OSA is a complex multifactorial disease with the interaction of multiple genes and environmental factors, leading to interindividual variability in the development of OSA. It is becoming increasingly clear that m6A modification plays an important regulatory role in the occurrence, development and prognosis of many human diseases (Tong et al., 2018; Zhang et al., 2020). However, researches of m6A regulators in OSA are still in its infancy. Satu Strausz conducted a large-scale genome-wide association study and found that rs9937053 near FTO associated with OSA (Strausz et al., 2021), which provided a great insight to investigate the relationship between m6A regulators and OSA. Herein, for the first time, we systematically investigate the impact of the m6A regulators in severe OSA, and a series of analyses were carried out to provide novel directions for the future studies and potentially effective therapies.

First of all, we compared the expression profiles between severe OSA patients and normal healthy subjects. The results showed that one “erasers” (ALKBH5), one “writers” (KIAA1429) and five “readers” (HNRNPA2B1, YTHDF2, FMR1, IGF2BP1 and IGF2BP3) m6A regulators statistically differentially expressed among 23 m6A regulators, implying their possible functional importance in severe OSA. Since m6A regulators have been showing have expression correlations or protein-protein interactions properties (Zhang et al., 2021), we further screened the correlations between m6A regulators and found that m6A regulators associated with each other quite differently in severe OSA and normal subjects. Especially, ZC3H13 and ALKBH5 were positively correlated in the normal group but negatively correlated in patients, which indicated that m6A regulators jointly influenced the occurrence and progression of severe OSA. Recently, using machine learning methods are effective ways to decide the important features or variables related to outcome of interest. We identified seven severe OSA-related m6A regulators by adopting LASSO and SVM algorithms, including HNRNPA2B1, METTL3, KIAA1429, YTHDF2, FMR1, IGF2BP1 and IGF2BP3. Among them, IGF2BP3 could be used to distinguish severe OSA patients from normal people in both training set and verification set GSE38792, which might be used as a diagnostic marker of severe OSA in the future.

Secondly, the general definition of OSA is classified according to its degree of AHI (mild, moderate, and severe), and they did not subdivide into more subclusters despite of the heterogeneity of patients. In this study, we used unsupervised consensus clustering for severe OSA based on the severe OSA-related m6A expression. The results showed that 24 severe OSA patients can be clustered into three subtypes with different expression patterns of m6A regulators, which proves the importance of m6A regulators in severe OSA again. Some studies have revealed that m6A regulators were tightly related to immune regulation (Li et al., 2017), so we speculate that the immune microenvironment characteristics of different severe OSA subtypes are also different. Our findings showed that the immune response in the RNA modification pattern of cluster3 was more active because it has the highest infiltrating level of M0 Macrophages and an increased trend infiltrating level of M2 Macrophages among three subclusters. Previously, Meng Qin Ge found that intermittent hypoxia (IH) could induces a pro-inflammatory phenotype of the subcutaneous adipose tissue with M1 macrophage polarization (Ge et al., 2019; Schaefer et al., 2017). Here, we showed that there is indeed a subcluster of severe OSA with obvious activation of M2 macrophages, which promote a better understanding of immune microenvironment of severe OSA. HLA genes and MHCs have been extensively studied due to their crucial role in immune responses. Suleiman M Momany found that HLA-DQB1*0602 allele was significantly associated with OSA (Momany et al., 2017). However, relationships between other HLA genes and severe OSA have not been studied. In this study, we observed eight HLA genes expression levels were upregulated in subcluster3. The results we have obtained shed new light on the immune properties of OSA, the classification strategy for severe OSA revealed a better understanding of the underlying regulatory mechanisms.

Thirdly, to further demonstrate the potential pathogenesis mechanisms involved in severe OSA, we explored the biological function of dysregulated genes by pairwise comparisons among different m6A patterns, as well as subclusters’ representative genes for each cluster. We found that three clusters have both unique and shared biological functions. The common enriched terms of these clusters were related to mitochondrial energy metabolism processes and multiple neurodegeneration diseases, which had been reported to accelerate the onset and development of OSA. For instance, chronic repetitive hypoxia in OSA caused dysfunctional mitochondria, damage in the electron transport chain (Lee et al., 2012; Zhou et al., 2016), and then could trigger neuron injury especially in the hippocampus and cerebral cortex regions (Zhou et al., 2016), which may be closely related to cognitive impairment. Additionally, reduced genioglossus muscle tone that controlled by the hypoglossal nerve (Perger and Taranto-Montemurro, 2021) is regarded as one of the major causes of OSA. Furtherly, we build the protein-protein interaction by using representative genes of each cluster. Genes that are associated with similar phenotypes are in proximity to each other in PPI networks (Kong et al., 2020), and the module analysis filtered out several hub genes. We also analyzed and visualized the miRNA-TF coregulatory network to understand the regulatory mechanism of hub genes at the transcriptional and post-transcriptional levels. However, very few studies have evaluated the relationship between these genes and OSA. The networks we established could provide novel clues for exploring the underlying regulatory mechanisms of key m6A regulators in the formation of OSA.

Finally, since there is still a lack of pharmacotherapy for OSA, specific small molecular compounds potentially acting on severe OSA were analyzed. The results showed that OSA subclusters were different in response to different small molecule drugs, highlighting the need for more targeted, subcluster-specific treatment. We found that ATPase inhibitors can aggravate the changes in genes expression of all severe OSA subtypes, while application of cyclooxygenase inhibitors and histamine receptor antagonists are conducive to reversing these changes. Different compounds act on adrenergic receptor antagonists having different responses to severe OSA patients and should be chosen carefully in the future. Very interestingly, our findings were consistent with previous research. Recently, a study of mice experiment has shown that histaminergic H3 receptor antagonists could partly ameliorate the negative effects on the hypoglossal nucleus and tuberomammillary nucleus caused by CIH (Xie et al., 2021). Abdulaziz A Alzahrani demonstrated that β-adrenoceptor blockade propranolol decreased respiratory frequency and abolished the CIH-mediated increase in vascular sympathetic nerve density. Similarly, several studies have shown that cyclooxygenase inhibitors can attenuate inflammatory responses by decreasing oxidative stress (Cheng et al., 2021). For example, Tissot Low showed that IH-induced lung inflammation could be completely abolished by daily intraperitoneal injection of ibuprofen (a cyclooxygenase inhibitor) (Cheng et al., 2021). Therefore, we speculate that cyclooxygenase inhibitors can improve OSA by inhibiting oxidative stress which needs to be demonstrated experimentally. Moreover, patients with OSA always have mitochondrial function damage (Yan et al., 2021) and inhibition of ATPase activities may have adverse effects. These studies have proved the validity of this bioinformatics analysis, and our results might facilitate the development of new and better therapies for severe OSA. Additionally, it was intriguing to note that several studies started shedding light on suitable drugs for OSA patients through clinical trials. Two randomized, placebo-controlled, double-blind crossover trials showed that the noradrenergic agent combined with the anti-muscarinic hyoscine could greatly reduce OSA severity (Lim et al., 2021; Taranto-Montemurro et al., 2019) by improving the measures of upper airway collapsibility. Ludovico Messineo and colleagues found that addition of zolpidem to combination therapy with atomoxetine-oxybutynin could even improve the sleep qualities of the OSA patients (Messineo et al., 2021). These studies have intervened from the activation of pharyngeal muscle to achieve the purpose of treatment, while nowadays transcriptomic data has been used to construct disease-drug correlations, which may lead to new drug repositioning theories (Sirota et al., 2011). Collectively, our work provides novel ideas for future clinical therapies or adjuvant treatment.

Several limitations should be acknowledged. First of all, this article is based on the bioinformatics analysis, which could provide a novel direction and ideas for future research, however, further experiments should be conducted to explore the specific molecular mechanisms. Next, this study only included severe OSA patients and the samples size is small, we tried to merge other GEO data sets but failed because of different chip platforms or tissue sources. Future studies can expand the sample size and contain patients with different conditions for typing in order to achieve a better understanding of the mechanisms underlying OSA. Besides, we are unable to obtain detailed clinical data of patients so we cannot evaluate the clinical symptoms and complications of patients with different subtypes.

## Conclusion

In conclusion, our study is the first one to investigate the crucial role of m6A methylation in severe OSA. We found that different m6A patterns in severe OSA have distinct immune microenvironment infiltration characterization and underlying molecular mechanisms, it fills the gap in the epigenetics of severe OSA, and opens up a new direction for carrying out more research in this field. Furthermore, the drugs and corresponding mechanisms were predicted logically as they were derived through analysis of DEGs between severe OSA and controls, which are important for pharmacotherapy development for OSA.

## Data Availability

Publicly available datasets were analyzed in this study. This data can be found here: https://www.ncbi.nlm.nih.gov/geo/GSE135917 and GSE38792.

## References

[B1] Barrett T. Troup D. B. Wilhite S. E. Ledoux P. Rudnev D. Evangelista C. (2007). NCBI GEO: Mining Tens of Millions of Expression Profiles-Ddatabase and Tools Update. Nucleic Acids Res. 35, D760–D765. 10.1093/nar/gkl887 17099226 PMC1669752

[B2] Chao Y. Shang J. Ji W. (2020). ALKBH5-m6A-FOXM1 Signaling axis Promotes Proliferation and Invasion of Lung Adenocarcinoma Cells under Intermittent Hypoxia. Biochem. Biophysical Res. Commun. 521, 499–506. 10.1016/j.bbrc.2019.10.145 31677788

[B3] Chen X.-Y. Zhang J. Zhu J.-S. (2019a). The Role of m6A RNA Methylation in Human Cancer. Mol. Cancer 18, 103. 10.1186/s12943-019-1033-z 31142332 PMC6540575

[B4] Chen Y. C. Hsu P. Y. Hsiao C. C. Lin M. C. (2019b). Epigenetics: A Potential Mechanism Involved in the Pathogenesis of Various Adverse Consequences of Obstructive Sleep Apnea. Int. J. Mol. Sci. 20. 10.3390/ijms20122937 PMC6627863 31208080

[B5] Cheng X. Zhao L. Ke T. Wang X. Cao L. Liu S. (2021). Celecoxib Ameliorates Diabetic Neuropathy by Decreasing Apoptosis and Oxidative Stress in Dorsal Root Ganglion Neurons via the miR-155/COX-2 axis. Exp. Ther. Med. 22, 825. 10.3892/etm.2021.10257 34149871 PMC8200812

[B6] Cortese R. Almendros I. Wang Y. Gozal D. (2015). Tumor Circulating DNA Profiling in Xenografted Mice Exposed to Intermittent Hypoxia. Oncotarget 6, 556–569. 10.18632/oncotarget.2785 25415227 PMC4381615

[B7] Davis S. Meltzer P. S. (2007). GEOquery: a Bridge between the Gene Expression Omnibus (GEO) and BioConductor. Bioinformatics 23, 1846–1847. 10.1093/bioinformatics/btm254 17496320

[B8] Deng X. Su R. Feng X. Wei M. Chen J. (2018). Role of N6-Methyladenosine Modification in Cancer. Curr. Opin. Genet. Development 48, 1–7. 10.1016/j.gde.2017.10.005 PMC5869081 29040886

[B9] Ge M. Q. Yeung S. C. Mak J. C. W. Ip M. S. M. (2019). Differential Metabolic and Inflammatory Responses to Intermittent Hypoxia in Substrains of Lean and Obese C57BL/6 Mice. Life Sci. 238, 116959. 10.1016/j.lfs.2019.116959 31628916

[B10] Gharib S. A. Hayes A. L. Rosen M. J. Patel S. R. (2013). A Pathway-Based Analysis on the Effects of Obstructive Sleep Apnea in Modulating Visceral Fat Transcriptome. Sleep 36, 23–30. 10.5665/sleep.2294 23288968 PMC3524507

[B11] Gharib S. A. Hurley A. L. Rosen M. J. Spilsbury J. C. Schell A. E. Mehra R. (2020). Obstructive Sleep Apnea and CPAP Therapy Alter Distinct Transcriptional Programs in Subcutaneous Fat Tissue. Sleep 43. 10.1093/sleep/zsz314 PMC7294406 31872261

[B12] Gleadhill I. C. Schwartz A. R. Schubert N. Wise R. A. Permutt S. Smith P. L. (1991). Upper Airway Collapsibility in Snorers and in Patients with Obstructive Hypopnea and Apnea. Am. Rev. Respir. Dis. 143, 1300–1303. 10.1164/ajrccm/143.6.1300 2048817

[B13] Hamada S. Tatsumi S. Kobayashi Y. Yasuba H. (2017). Nasal Nitric Oxide Improved by Continuous Positive Airway Pressure Therapy for Upper Airway Inflammation in Obstructive Sleep Apnea. Sleep Breath 21, 405–410. 10.1007/s11325-016-1431-z 27837378

[B14] Hänzelmann S. Castelo R. Guinney J. (2013). GSVA: Gene Set Variation Analysis for Microarray and RNA-Seq Data. BMC Bioinformatics 14, 7. 10.1186/1471-2105-14-7 23323831 PMC3618321

[B15] Javaheri S. Barbe F. Campos-Rodriguez F. Dempsey J. A. Khayat R. Javaheri S. (2017). Sleep Apnea. J. Am. Coll. Cardiol. 69, 841–858. 10.1016/j.jacc.2016.11.069 28209226 PMC5393905

[B16] Karthiya R. Khandelia P. (2020). m6A RNA Methylation: Ramifications for Gene Expression and Human Health. Mol. Biotechnol. 62, 467–484. 10.1007/s12033-020-00269-5 32840728

[B17] Kong J. Lee H. Kim D. Han S. K. Ha D. Shin K. (2020). Network-based Machine Learning in Colorectal and Bladder Organoid Models Predicts Anti-cancer Drug Efficacy in Patients. Nat. Commun. 11, 5485. 10.1038/s41467-020-19313-8 33127883 PMC7599252

[B18] Lamb J. Crawford E. D. Peck D. Modell J. W. Blat I. C. Wrobel M. J. (2006). The Connectivity Map: Using Gene-Expression Signatures to Connect Small Molecules, Genes, and Disease. Science 313, 1929–1935. 10.1126/science.1132939 17008526

[B19] Lee J. Giordano S. Zhang J. (2012). Autophagy, Mitochondria and Oxidative Stress: Cross-Talk and Redox Signalling. Biochem. J. 441, 523–540. 10.1042/bj20111451 22187934 PMC3258656

[B20] Li H.-B. Tong J. Zhu S. Batista P. J. Duffy E. E. Zhao J. (2017). m6A mRNA Methylation Controls T Cell Homeostasis by Targeting the IL-7/STAT5/SOCS Pathways. Nature 548, 338–342. 10.1038/nature23450 28792938 PMC5729908

[B21] Lim R. Messineo L. Grunstein R. R. Carberry J. C. Eckert D. J. (2021). The Noradrenergic Agent Reboxetine Plus the Antimuscarinic Hyoscine Butylbromide Reduces Sleep Apnoea Severity: a Double‐blind, Placebo‐controlled, Randomised Crossover Trial. J. Physiol. 599, 4183–4195. 10.1113/jp281912 34174090

[B22] Liu Z. P. Wu C. Miao H. Wu H. (2015). RegNetwork: an Integrated Database of Transcriptional and post-transcriptional Regulatory Networks in Human and Mouse. Database (Oxford) 2015, bav095. 10.1093/database/bav095 26424082 PMC4589691

[B23] Luo W. Brouwer C. (2013). Pathview: an R/Bioconductor Package for Pathway-Based Data Integration and Visualization. Bioinformatics 29, 1830–1831. 10.1093/bioinformatics/btt285 23740750 PMC3702256

[B24] Malta T. M. Sokolov A. Gentles Aj. Burzykowski T. Poisson L. Weinstein J. N. (2018). Machine Learning Identifies Stemness Features Associated with Oncogenic Dedifferentiation. Cell 173, 338–354. e15. 10.1016/j.cell.2018.03.034 29625051 PMC5902191

[B25] Marin J. M. Artal J. Martin T. Carrizo S. J. Andres M. Martin-Burriel I. (2014). Epigenetics Modifications and Subclinical Atherosclerosis in Obstructive Sleep Apnea: The EPIOSA Study. BMC Pulm. Med. 14, 114. 10.1186/1471-2466-14-114 25016368 PMC4107483

[B26] Mathiyalagan P. Adamiak M. Mayourian J. Sassi Y. Liang Y. Agarwal N. (2019). FTO-dependent N 6 -Methyladenosine Regulates Cardiac Function during Remodeling and Repair. Circulation 139, 518–532. 10.1161/circulationaha.118.033794 29997116 PMC6400591

[B27] McNicholas W. T. (2009). Obstructive Sleep Apnea and Inflammation. Prog. Cardiovasc. Dis. 51, 392–399. 10.1016/j.pcad.2008.10.005 19249445

[B28] Mehrtash M. Bakker J. P. Ayas N. (2019). Predictors of Continuous Positive Airway Pressure Adherence in Patients with Obstructive Sleep Apnea. Lung 197, 115–121. 10.1007/s00408-018-00193-1 30617618

[B29] Messineo L. Carter S. G. Taranto‐Montemurro L. Chiang A. Vakulin A. Adams R. J. (2021). Addition of Zolpidem to Combination Therapy with Atomoxetine‐oxybutynin Increases Sleep Efficiency and the Respiratory Arousal Threshold in Obstructive Sleep Apnoea: A Randomized Trial. Respirology 26, 878–886. 10.1111/resp.14110 34164887

[B30] Meyer D. Dimitriadou E. Hornik K. Weingessel A. Leisch F. Chang C. C. (2015). e1071: Misc Functions of the Department of Statistics Probability Theory Group (Formerly: E1071). Vienna, Austria: TU Wien.

[B31] Momany S. M. Al-Qatarneh T. A. Khader Y. S. Abu-Harfeil N. M. Daoud A. K. Mahasneh A. A. (2017). The Association of HLA-Dqb1*0602 but Not HLA-Drb1*15 with Obstructive Sleep Apnea. Cim 40, E167–e175. 10.25011/cim.v40i4.28494 28875928

[B32] Newman A. M. Steen C. B. Liu C. L. Gentles A. J. Chaudhuri A. A. Scherer F. (2019). Determining Cell Type Abundance and Expression from Bulk Tissues with Digital Cytometry. Nat. Biotechnol. 37, 773–782. 10.1038/s41587-019-0114-2 31061481 PMC6610714

[B33] Peppard P. E. Young T. Barnet J. H. Palta M. Hagen E. W. Hla K. M. (2013). Increased Prevalence of Sleep-Disordered Breathing in Adults. Am. J. Epidemiol. 177, 1006–1014. 10.1093/aje/kws342 23589584 PMC3639722

[B34] Perger E. Taranto-Montemurro L. (2021). Upper Airway Muscles: Influence on Obstructive Sleep Apnoea Pathophysiology and Pharmacological and Technical Treatment Options. Curr. Opin. Pulm. Med. 27, 505–513. 10.1097/mcp.0000000000000818 34431788

[B35] Ritchie M. E. Phipson B. Wu D. Hu Y. Law C. W. Shi W. (2015). Limma powers Differential Expression Analyses for RNA-Sequencing and Microarray Studies. Nucleic Acids Res. 43, e47. 10.1093/nar/gkv007 25605792 PMC4402510

[B36] Robin X. Turck N. Hainard A. Tiberti N. Lisacek F. Sanchez J.-C. (2011). pROC: an Open-Source Package for R and S+ to Analyze and Compare ROC Curves. BMC Bioinformatics 12, 77. 10.1186/1471-2105-12-77 21414208 PMC3068975

[B37] Roundtree I. A. Evans M. E. Pan T. He C. (2017). Dynamic RNA Modifications in Gene Expression Regulation. Cell 169, 1187–1200. 10.1016/j.cell.2017.05.045 28622506 PMC5657247

[B38] Schaefer E. Wu W. Mark C. Yang A. Digiacomo E. Carlton-Smith C. (2017). Intermittent Hypoxia Is a Proinflammatory Stimulus Resulting in IL-6 Expression and M1 Macrophage Polarization. Hepatol. Commun. 1, 326–337. 10.1002/hep4.1045 29404462 PMC5721395

[B39] Simon N. Friedman J. Hastie T. Tibshirani R. (2011). Regularization Paths for Cox's Proportional Hazards Model via Coordinate Descent. J. Stat. Softw. 39, 1–13. 10.18637/jss.v039.i05 PMC4824408 27065756

[B40] Sirota M. Dudley Jt. Kim J. Chiang Ap. Morgan Aa. Sweet-Cordero A. (2011). Discovery and Preclinical Validation of Drug Indications Using Compendia of Public Gene Expression Data. Sci. Transl Med. 3, 96ra77. 10.1126/scitranslmed.3001318 PMC3502016 21849665

[B41] Strausz S. Ruotsalainen S. Ollila Hm. Karjalainen J. Kiiskinen T. Reeve M. (2021). Genetic Analysis of Obstructive Sleep Apnoea Discovers a strong Association with Cardiometabolic Health. Eur. Respir. J. 57. 10.1183/13993003.03091-2020 33243845

[B42] Subramanian A. Narayan R. Corsello S. M. Peck D. D. Natoli T. E. Lu X. (2017). A Next Generation Connectivity Map: L1000 Platform and the First 1,000,000 Profiles. Cell 171, 1437–1452. 10.1016/j.cell.2017.10.049 29195078 PMC5990023

[B43] Sullivan C. Berthon-Jones M. Issa F. Eves L. (1981). Reversal of Obstructive Sleep Apnoea by Continuous Positive Airway Pressure Applied through the Nares. The Lancet 317, 862–865. 10.1016/s0140-6736(81)92140-1 6112294

[B44] Szklarczyk D. Gable A. L. Lyon D. Junge A. Wyder S. Huerta-Cepas J. (2019). STRING V11: Protein-Protein Association Networks with Increased Coverage, Supporting Functional Discovery in Genome-wide Experimental Datasets. Nucleic Acids Res. 47, D607–d613. 10.1093/nar/gky1131 30476243 PMC6323986

[B45] Taranto-Montemurro L. Messineo L. Sands S. A. Azarbarzin A. Marques M. Edwards B. A. (2019). The Combination of Atomoxetine and Oxybutynin Greatly Reduces Obstructive Sleep Apnea Severity. A Randomized, Placebo-Controlled, Double-Blind Crossover Trial. Am. J. Respir. Crit. Care Med. 199, 1267–1276. 10.1164/rccm.201808-1493oc 30395486 PMC6519859

[B46] Tong J. Flavell R. A. Li H.-B. (2018). RNA m6A Modification and its Function in Diseases. Front. Med. 12, 481–489. 10.1007/s11684-018-0654-8 30097961

[B47] Van D. M. L. Hinton G. (2008). Visualizing High-Dimensional Data Using T-SNE. J. Machine Learn. Res. 9, 2579–2605.

[B48] Wei T. Simko V. J. M. M. Report M. W. (2013). Corrplot: Visualization of a Correlation Matrix, R Package, 145–151. Available at: https://github.com/taiyun/corrplot .

[B49] Whelton P. K. Carey R. M. Aronow W. S. Casey D. E. Jr. Collins K. J. Dennison Himmelfarb C. (2018). 2017 ACC/AHA/AAPA/ABC/ACPM/AGS/APhA/ASH/ASPC/NMA/PCNA Guideline for the Prevention, Detection, Evaluation, and Management of High Blood Pressure in Adults: A Report of the American College of Cardiology/American Heart Association Task Force on Clinical Practice Guidelines. J. Am. Coll. Cardiol. 71, e127–e248. 10.1016/j.jacc.2017.11.006 29146535

[B50] Wilkerson M. D. Hayes D. N. (2010). ConsensusClusterPlus: a Class Discovery Tool with Confidence Assessments and Item Tracking. Bioinformatics 26, 1572–1573. 10.1093/bioinformatics/btq170 20427518 PMC2881355

[B51] Xia J. Benner M. J. Hancock R. E. W. (2014). NetworkAnalyst - Integrative Approaches for Protein-Protein Interaction Network Analysis and Visual Exploration. Nucleic Acids Res. 42, W167–W174. 10.1093/nar/gku443 24861621 PMC4086107

[B52] Xia J. Gill E. E. Hancock R. E. W. (2015). NetworkAnalyst for Statistical, Visual and Network-Based Meta-Analysis of Gene Expression Data. Nat. Protoc. 10, 823–844. 10.1038/nprot.2015.052 25950236

[B53] Xie L. Wu Q. Hu W. Wu X. Xiang G. Hao S. (2021). Impact of Histaminergic H3 Receptor Antagonist on Hypoglossal Nucleus in Chronic Intermittent Hypoxia Conditions. Psychopharmacology 238, 121–131. 10.1007/s00213-020-05663-0 32964244

[B54] Yan Y. R. Zhang L. Lin Y. N. Sun X. W. Ding Y. J. Li N. (2021). Chronic Intermittent Hypoxia-Induced Mitochondrial Dysfunction Mediates Endothelial Injury via the TXNIP/NLRP3/IL-1β Signaling Pathway. Free Radic. Biol. Med. 165, 401–410. 10.1016/j.freeradbiomed.2021.01.053 33571641

[B55] Younes M. (2008). Role of Respiratory Control Mechanisms in the Pathogenesis of Obstructive Sleep Disorders. J. Appl. Physiol. (1985) 105, 1389–1405. 10.1152/japplphysiol.90408.2008 18787092

[B56] Yu G. Wang L.-G. Han Y. He Q.-Y. (2012). clusterProfiler: an R Package for Comparing Biological Themes Among Gene Clusters. OMICS: A J. Integr. Biol. 16, 284–287. 10.1089/omi.2011.0118 PMC3339379 22455463

[B57] Zhang B. Wu Q. Li B. Wang D. Wang L. Zhou Y. L. (2020). m6A Regulator-Mediated Methylation Modification Patterns and Tumor Microenvironment Infiltration Characterization in Gastric Cancer. Mol. Cancer 19, 53. 10.1186/s12943-020-01170-0 32164750 PMC7066851

[B58] Zhang X. Zhang S. Yan X. Shan Y. Liu L. Zhou J. (2021). m6A Regulator‐mediated RNA Methylation Modification Patterns Are Involved in Immune Microenvironment Regulation of Periodontitis. J. Cell Mol Med 25, 3634–3645. 10.1111/jcmm.16469 33724691 PMC8034465

[B59] Zhou L. Chen P. Peng Y. Ouyang R. (2016). Role of Oxidative Stress in the Neurocognitive Dysfunction of Obstructive Sleep Apnea Syndrome. Oxid Med. Cell Longev 2016, 9626831. 10.1155/2016/9626831 27774119 PMC5059616

