# Supplementary material for: Comprehensive Analysis of N6-Methyladenosine Regulators in the Subcluster Classification and Drug Candidates Prediction of Severe Obstructive Sleep Apnea
Source: Front Genet. 2022 Apr 26;13:862972. doi: 10.3389/fgene.2022.862972 (PMC9086428; doi:10.3389/fgene.2022.862972)
Supplement: Supplementary file 1 [file DataSheet1.PDF]

Supplementary Material

1 Supplementary Figures and Tables

1.1 Supplementary Figures

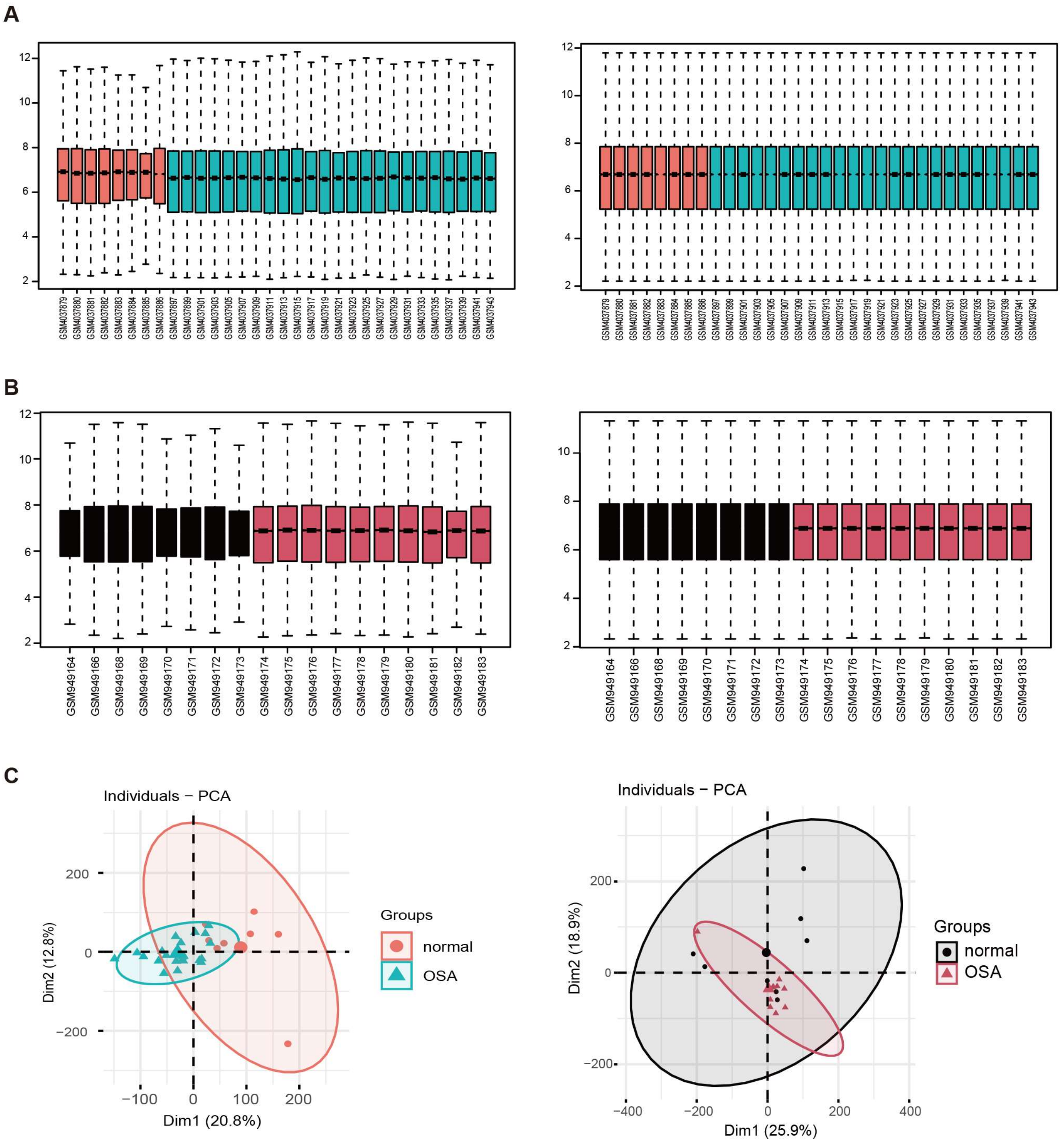

Supplementary Figure 1. Processing of the expression profiles. (A) GSE135917 data before normalization (left) and after normalization (right). (B) GSE38792 data before normalization (left)

and after normalization (right). (C) Principal component analysis for the transcriptome profiles of GSE135917 (left) and GSE38792 (right).

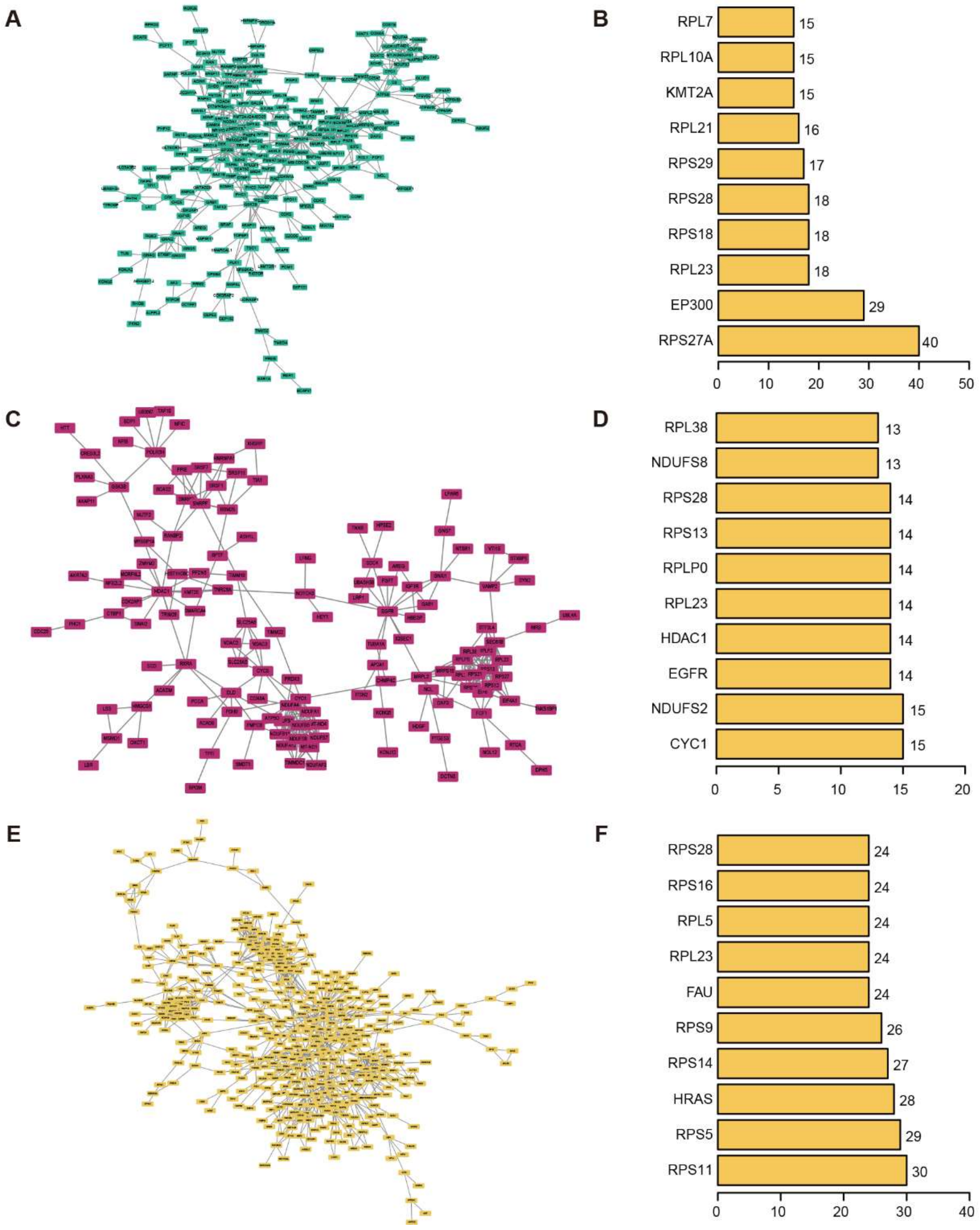

Supplementary Figure 2. The protein-protein interaction networks for each OSA subtypes. Each node represents a gene and each edge represents one interaction pair. The protein-protein interaction networks of representative genes for cluster1 (A), cluster2 (C) and cluster3 (E). The top 10 genes with the most interactions and the number of protein-protein interaction pairs for cluster1 (B), cluster2 (D) and cluster3 (F).

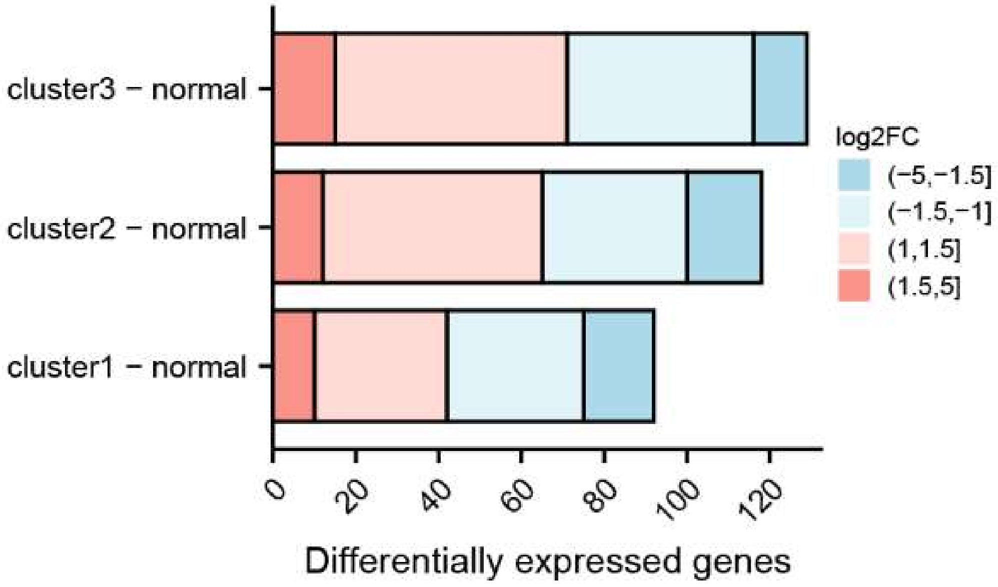

Supplementary Figure 3. Differentially expressed genes between severe OSA subtypes and normal control group. The abscissa represents the number of differentially expressed genes.
